# Correction: A common methodological phylogenomics framework for intra-patient heteroplasmies to infer SARS-CoV-2 sublineages and tumor clones

**DOI:** 10.1186/s12864-022-08959-x

**Published:** 2023-01-05

**Authors:** Filippo Utro, Chaya Levovitz, Kahn Rhrissorrakrai, Laxmi Parida

**Affiliations:** IBM Research, T.J. Watson Research Center, Yorktown Heights, USA

**Correction: BMC Genomics 22, 518 (2021)**

**https://doi.org/10.1186/s12864-021-07660-9**

Following publication of the original article [1], it was reported that Figs. 4, 5 and 6 were published out of order, though the captions were correct. The figures for Figs. 4, 5 and 6 were originally published as Figs. 5, 6 and 4 respectively.

The correct figures are given in this Correction article and the original article [1] has been updated.

**Fig. 4:**
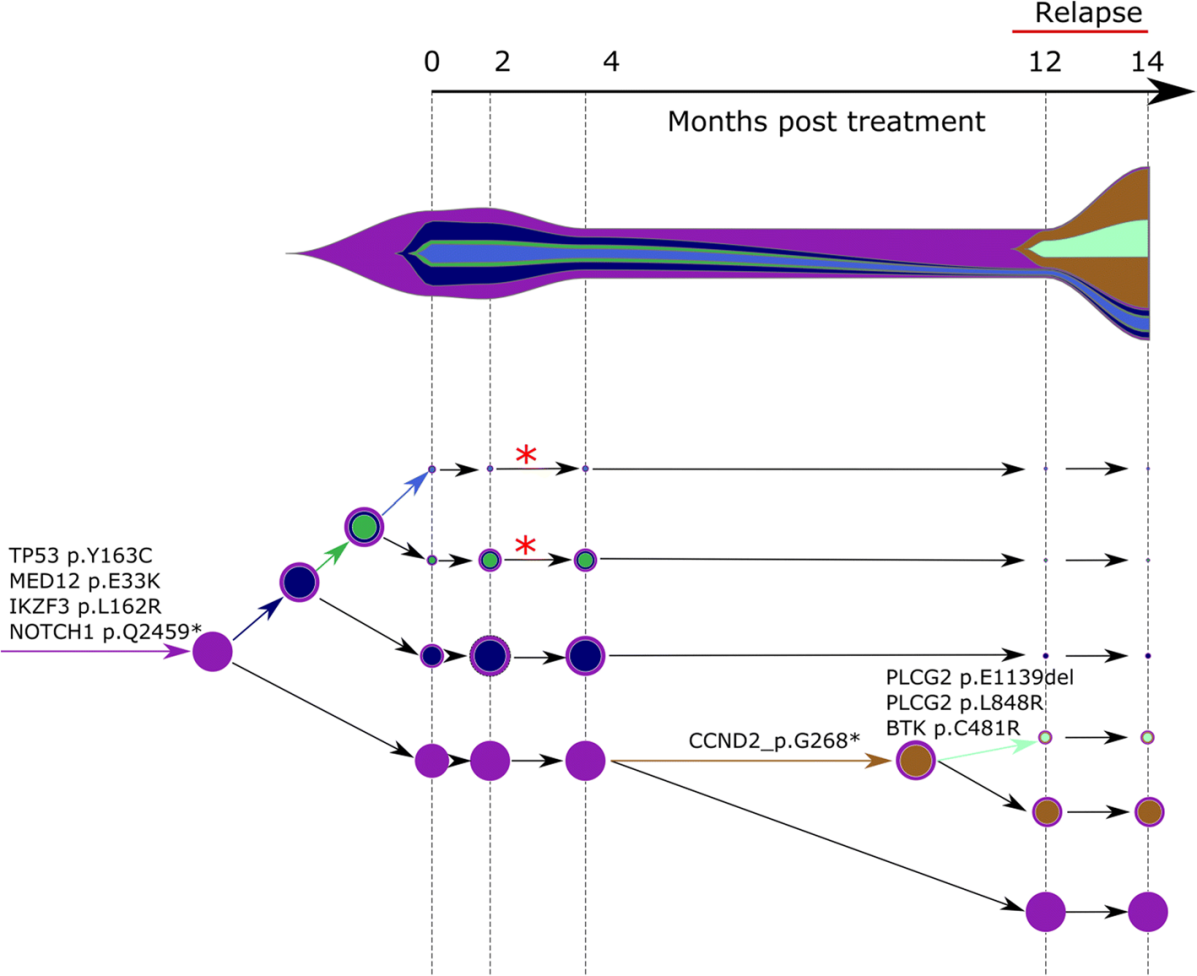
Concerti fishplot and tumor evolution tree **T** for patient CLL1 multi-time data. The fishplot width corresponds to approximate tumor size using ALC (absolute lymphocyte count) values. Clones are colored and sized proportionally to their prevalence. The corresponding tumor tree is aligned by timepoint and highlights to the birth of brown clone, which occurred prior to the annotation of clinical relapse. Node sizes correspond to prevalence. The edges of the **T** are labeled by the known cancer genes and the colors denote the distinct pseudoclones estimated by Concerti. Red asterisks indicates acquisition of new alterations to clone

**Fig. 5:**
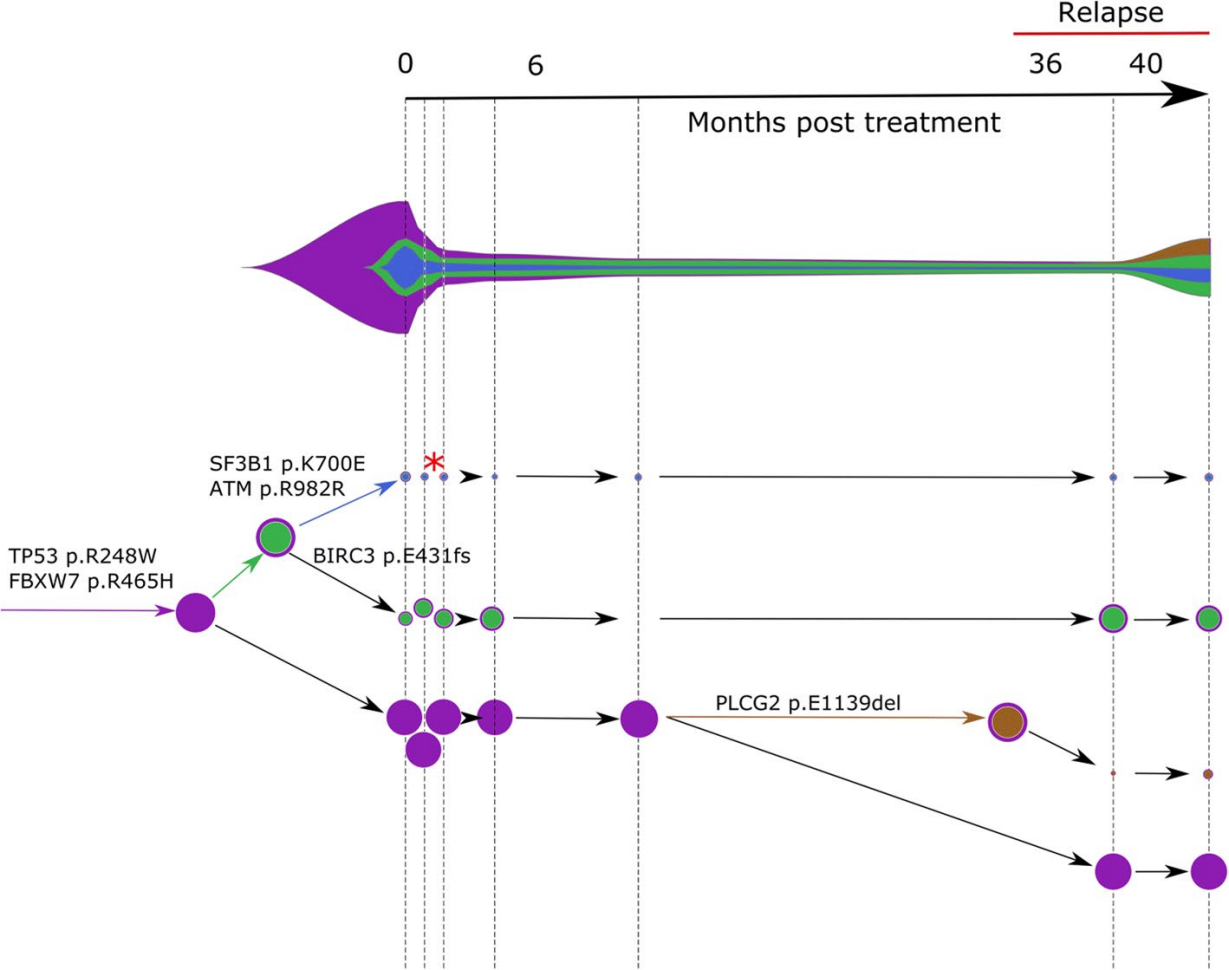
Concerti fishplot and tumor evolution tree **T** for patient CLL2 multi-time data. The fishplot width corresponds to approximate tumor size using ALC values. Clones are colored and sized proportionally to their prevalence. The corresponding tumor tree is aligned by timepoint and highlights to the birth of brown clone, which occurred prior to the annotation of clinical relapse. Node sizes correspond to prevalence. The edges of the **T** are labeled by the known cancer genes and the colors denote the distinct pseudoclones estimated by Concerti. Red asterisk indicates acquisition of new alterations to clone

**Fig. 6:**
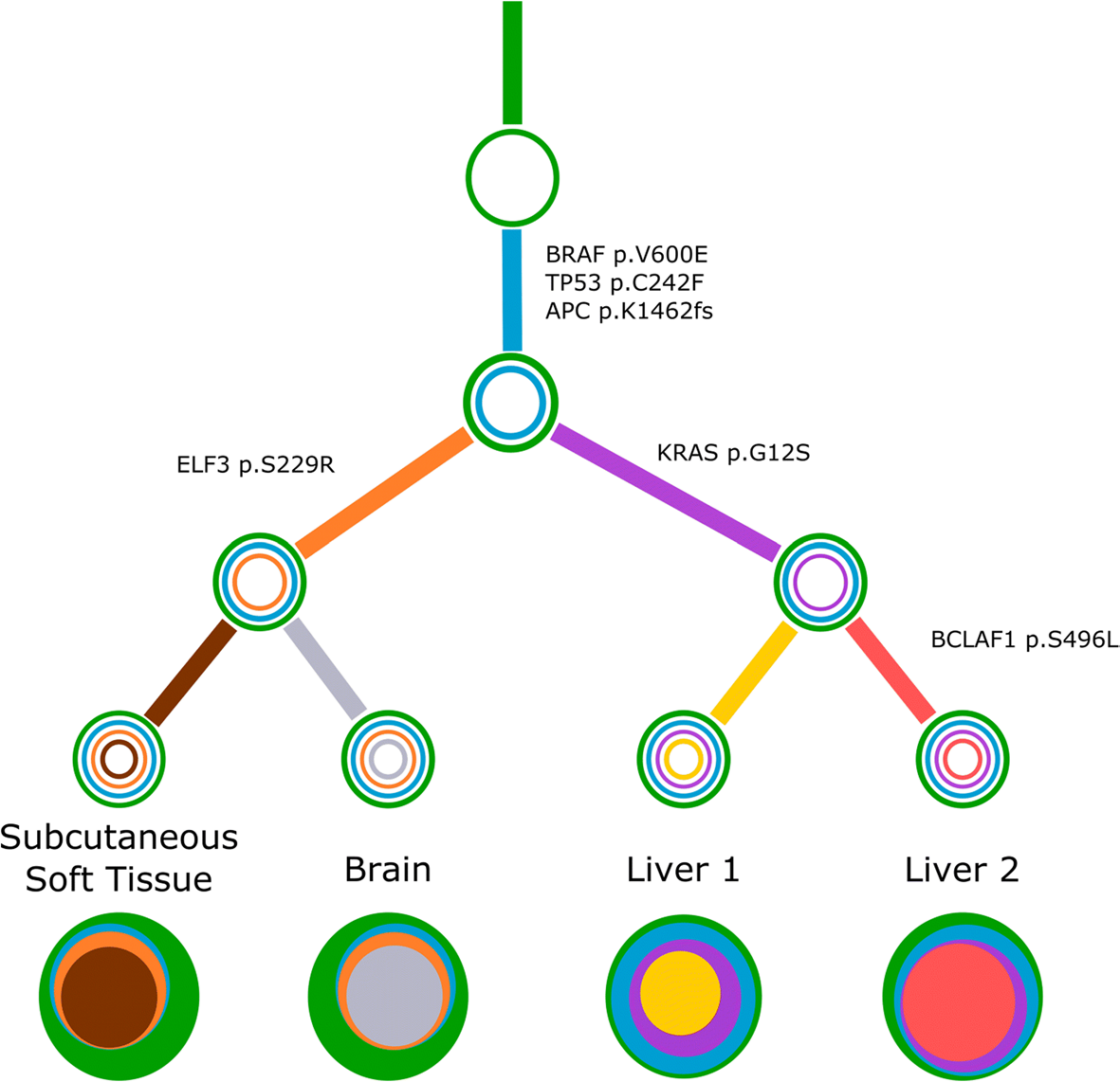
Concerti tumor evolution tree **T** for patient GI1. Tumor evolution tree **T** for colon cancer patient GI1 multi-site data. The edges of the **T** are labeled by the known cancer genes and the colors denote the distinct pseudoclones estimated by Concerti. Leaf nodes represent each of the distinct lesion sites. The single site trees ***τ*** are shown at the bottom as stacked discs and the sizes are proportional to the prevalence values
